# Fasting and High-Fat Diet Alter Histone Deacetylase Expression in the Medial Hypothalamus

**DOI:** 10.1371/journal.pone.0018950

**Published:** 2011-04-15

**Authors:** Hiromasa Funato, Satoko Oda, Junko Yokofujita, Hiroaki Igarashi, Masaru Kuroda

**Affiliations:** Department of Anatomy, Toho University School of Medicine, Tokyo, Japan; Brigham and Women's Hospital, Harvard Medical School, United States of America

## Abstract

Increasing attention is now being given to the epigenetic regulation of animal and human behaviors including the stress response and drug addiction. Epigenetic factors also influence feeding behavior and metabolic phenotypes, such as obesity and insulin sensitivity. In response to fasting and high-fat diets, the medial hypothalamus changes the expression of neuropeptides regulating feeding, metabolism, and reproductive behaviors. Histone deacetylases (HDACs) are involved in the epigenetic control of gene expression and alter behavior in response to a variety of environmental factors. Here, we examined the expression of HDAC family members in the medial hypothalamus of mice in response to either fasting or a high-fat diet. In response to fasting, *HDAC3* and *−4* expression levels increased while *HDAC10* and *−11* levels decreased. Four weeks on a high-fat diet resulted in the increased expression of *HDAC5* and *−8*. Moreover, fasting decreased the number of acetylated histone H3- and acetylated histone H4-positive cells in the ventrolateral subdivision of the ventromedial hypothalamus. Therefore, HDACs may be implicated in altered gene expression profiles in the medial hypothalamus under different metabolic states.

## Introduction

A growing number of studies have shown that environmental factors including stress, maternal care, and exposure to psychostimulants epigenetically modulate gene expression through chromosomal modification, resulting in altered behavior [1]–[3]. Consistent with the role of epigenetic control in a variety of behaviors, the time-scale of epigenetic regulation varies widely, from circadian changes of histone acetylation in the promoter regions of clock genes [4] to long-term memory formation [5].

Epigenetic factors also regulate feeding behavior and metabolic phenotypes such as obesity and insulin sensitivity. Many human and animal studies have shown that maternal metabolic abnormalities have long-term effects on the metabolic characteristics of offspring [6], [7]. In addition to maternal factors, it has been reported recently that paternal epigenetics influence pancreatic islet growth and glucose metabolism in female offspring [8].

Feeding behavior and body-weight homeostasis are regulated by a neural network within the hypothalamus and brain stem [9], [10]. The hypothalamic arcuate nucleus (ARH) contains two populations of neurons crucial for feeding behavior regulation. One of these populations expresses anorexigenic, α-melanocyte stimulating hormone processed from pro-opiomelanocortin (POMC), whereas the second population expresses orexigenic neuropeptides, including neuropeptide Y (NPY) and agouti-related peptide (AgRP). Both POMC neurons and NPY/AgRP-expressing neurons receive direct and indirect inputs from orexin neurons located in the lateral hypothalamic area (LHA) and send their fibers to the paraventricular hypothalamic nucleus (PVH) to regulate feeding behavior [11]. The ventromedial hypothalamic nucleus (VMH) has close fiber connections with the ARH, and is involved in body weight regulation [12], [13] as well as aggression and sexual behaviors [14]. In response to fasting and a high-fat diet, the expression levels of genes regulating feeding and reproductive behaviors in the hypothalamus are modified so that animals behave according to different energy needs [15]–[19].

The mechanisms by which gene expressions are modulated in the hypothalamus under different metabolic conditions have recently attracted considerable attention [20]–[22]. The histone deacetylase (HDAC) family is comprised of the following protein classes: class I (HDAC1, HDAC2, HDAC3, and HDAC8), class IIa (HDAC4, HDAC5, HDAC7, and HDAC9), class IIb (HDAC6 and HDAC10) and class IV (HDAC11)[23]–[25]. HDACs regulate histone acetylation in a sequence-specific and more global manner to repress, and in some cases enhance, gene transcription. Recently, HDACs have received increased attention due to the fact that they have been shown to play important roles in altered behavior in response to stress, chronic cocaine exposure, and energy metabolism [26], [27]. These findings suggest that the HDAC family may be involved in the changes in hypothalamic gene expression that occur under different metabolic conditions.

In the present study, we examined the expression levels of HDAC family members in the medial hypothalamus in response to fasting or a high-fat diet. We further performed immunohistochemical examination of histone acetylation within the medial hypothalamus.

## Results

**Figure 1:**
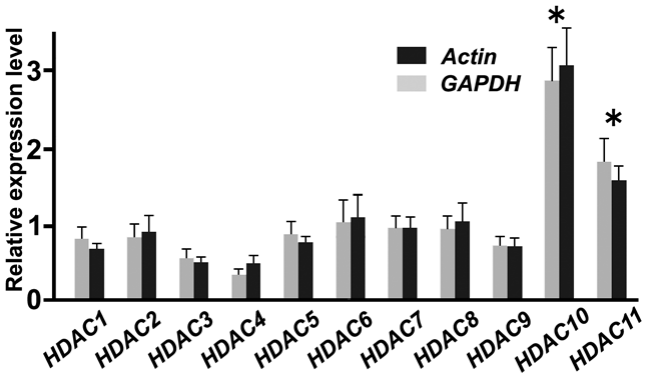
Expression of *HDACs* in the medial hypothalamus of fed mice. The medial hypothalamus of fed mice showed similar expression levels of *HDAC1, −2, −3, −4, −5, −6, −7, −8,* and *−9*. The expressions of *HDAC10* and *−11* were significantly higher than those of other HDACs. *HDAC* expression levels (n = 10) were normalized using *GAPDH* (gray bars) or*β-actin* expression levels (black bars). Reference gene selection did not affect the measurements of normalized *HDAC* levels. *HDAC* expression was also normalized based on the average of all *HDAC* levels. *P<0.05.

We first examined the expression of *HDAC* in the medial hypothalamus of fed adult mice. All *HDAC* transcripts, from *HDAC1* to *HDAC11*, were detected in the medial hypothalamus with differing levels of expression. The expressions of *HDAC10* and -*11* were significantly higher than those of other HDACs (Figure 1).

**Figure 2:**
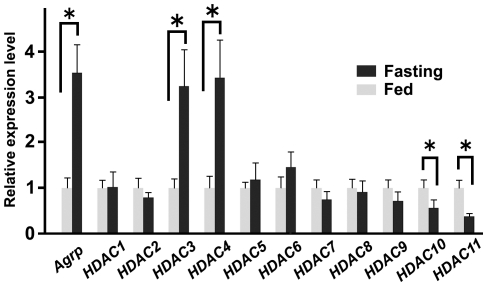
Fasting affects the expression of certain HDAC members family in the medial hypothalamus. After 16-hours of fasting, the expression levels of *HDAC3, −4,* and *Agrp* in the medial hypothalamus were increased, and *HDAC10* and −*11* levels decreased when compared with levels measured under fed conditions (8–10 mice per group). Data are presented as the expression level relative to the fed condition. *P<0.05.

We next examined whether fasting alters the expression of *HDAC* family members in the medial hypothalamus. After 16-hours of fasting, *HDAC3* and −*4* expression levels were significantly increased compared to those of normally-fed mice, whereas *HDAC10* and −*11* expressions were decreased (Figure 2). Fasting did not alter the expression of *HDAC1, −2, −5, −6, −7, −8* or, *−9*. Fasting also increased the expression of the orexigenic peptide *Agrp* as previously reported [19].

**Figure 3:**
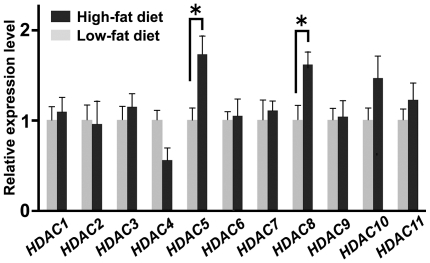
A high-fat diet affects the expression of certain HDAC family members in the medial hypothalamus. After 4-weeks on a high-fat diet, the expression levels of *HDAC5* and −*8* increased when compared to those measured from mice fed a low-fat diet (8–10 mice per group). Data are presented as an expression level relative to the low-fat diet condition. *P<0.05.

To further examine whether metabolic status affects the expression of *HDAC* genes in the medial hypothalamus, we determined *HDAC* expression levels in mice with high-fat diet-induced obesity. After 4-weeks of high-fat feeding, mice displayed a significantly larger body weight than mice fed a low-fat diet (high-fat diet n = 8, 7.2 g±0.7; low-fat diet n = 10, 2.2 g±0.8; p<0.01). Among all *HDACs* examined, a high-fat diet significantly increased the expression of *HDAC5* and −*8* (p<0.05; Figure 3). HDAC4 tended to decrease, but did not reach significance (p = 0.10).

**Figure 4:**
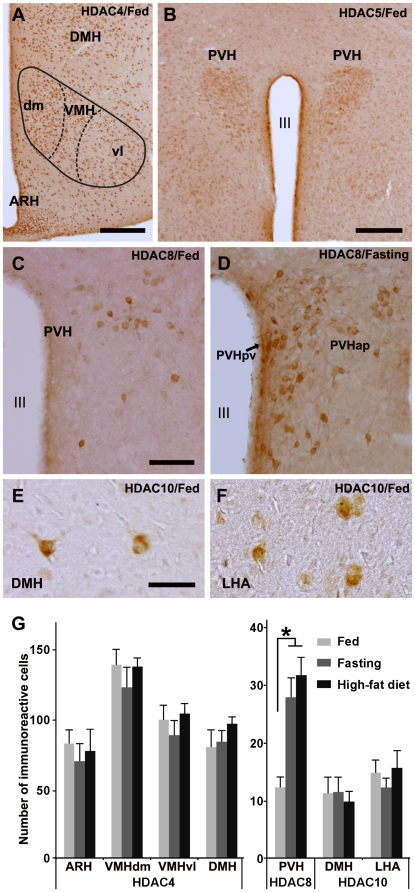
HDAC immunoreactivities in the medial hypothalamus. A) HDAC4-immunoreactive cells were found throughout the hypothalamus including the ARH, VMHdm, VMHvl, and DMH of fed mice. B) The PVH of fed mice was immunoreactive for HDAC5. C, D) HDAC8-positive cells were found in the anterior parvicellular and periventricular subdivisions of the PVH of fed (C) and fasting mice (D). E, F) A small number of HDAC10-immunoreactive cells were seen in the DMH (E) and LHA (F). G) Graphs showing the number of HDAC4, −8, and −10 positive cells in the nuclei of the medial hypothalamus. The number of HDAC8-positive cells in the PVH of fasting or high-fat fed mice was significantly larger than that of mice fed a low-fat diet (n = 5). Scale bars: 250 µm (A, B); 100 µm (C); 50 µm (E). *P<0.05. ARH: arcuate nucleus, dm: dorsomedial subdivision, DMH: dorsomedial hypothalamic nucleus, LHA: lateral hypothalamic area, PVH: paraventricular nucleus, PVHap: anterior parvicellular subdivion of PVH, PVHpv: periventricular subdivision of PVH, vl: ventrolateral subdivision, VMH: ventromedial hypothalamic nucleus, III: the third ventricle.

We next performed immunohistochemical analyses of HDAC proteins in the hypothalamus using antibodies against HDAC3, −4, −5, −8, −10, and −11. HDAC3-immunostaining showed weak and diffuse immunoreactivity throughout the gray mater. HDAC4-immunoreactive cells were found throughout the hypothalamus including the ARH, dorsomedial subdivision of VMH (VMHdm), ventrolateral subdivision of the VMH (VMHvl), and dorsomedial hypothalamic nucleus (DMH) of fed mice (Figure 4A). The numbers of HDAC4-immunoreactive cells in the ARH, VMHdm, VMHvl, and DMH of fed mice were similar to those of fasting or high-fat feeding mice (Figure 4G). The PVH of fed mice were weakly immunoreactive for HDAC5 (Figure 4B). The extent and intensity of HDAC5-immunoreactivity in the PVH of mice fed normal chow was similar to those of mice under fasting and high-fat diet feeding. Weakly HDAC5-immunoreactive cells were also found in the ARH and DMH. Small numbers of HDAC8-positive cells were found in the anterior parvicellular and periventricular subdivisions of the PVH (Figure 4C, D), VMHdm, and LHA. The cytoplasmic localization of HDAC8 is consistent with a previous report on HDAC8 in muscular cells [28]. No HDAC8-positive cells were found in the ARH or VMH. Both fasting and a high fat-diet increased the number of HDAC8-positive cells in the PVH (Figure 4C, D, G) but not in the VMHdm and LHA. A small number of HDAC10-immunoreactive cells were seen in the DMH and LHA (Figure 4E, F), but not in the PVH, VMH, or ARH of mice fed normal chow. HDAC10-immunoreactivities were observed in the cytoplasm and proximal portion of the dendrites (Figure 4E, F). The numbers of HDAC10-positive cells in the DMH and LHA of mice fed normal chow was similar to those of mice under fasting or a high-fat diet (Figure 4G). HDAC11-immunostaining showed weak cytoplasmic and nuclear staining with moderate staining in the ARH. However, it was not possible to examine differential expression levels or to count the number of positive cells.

**Figure 5:**
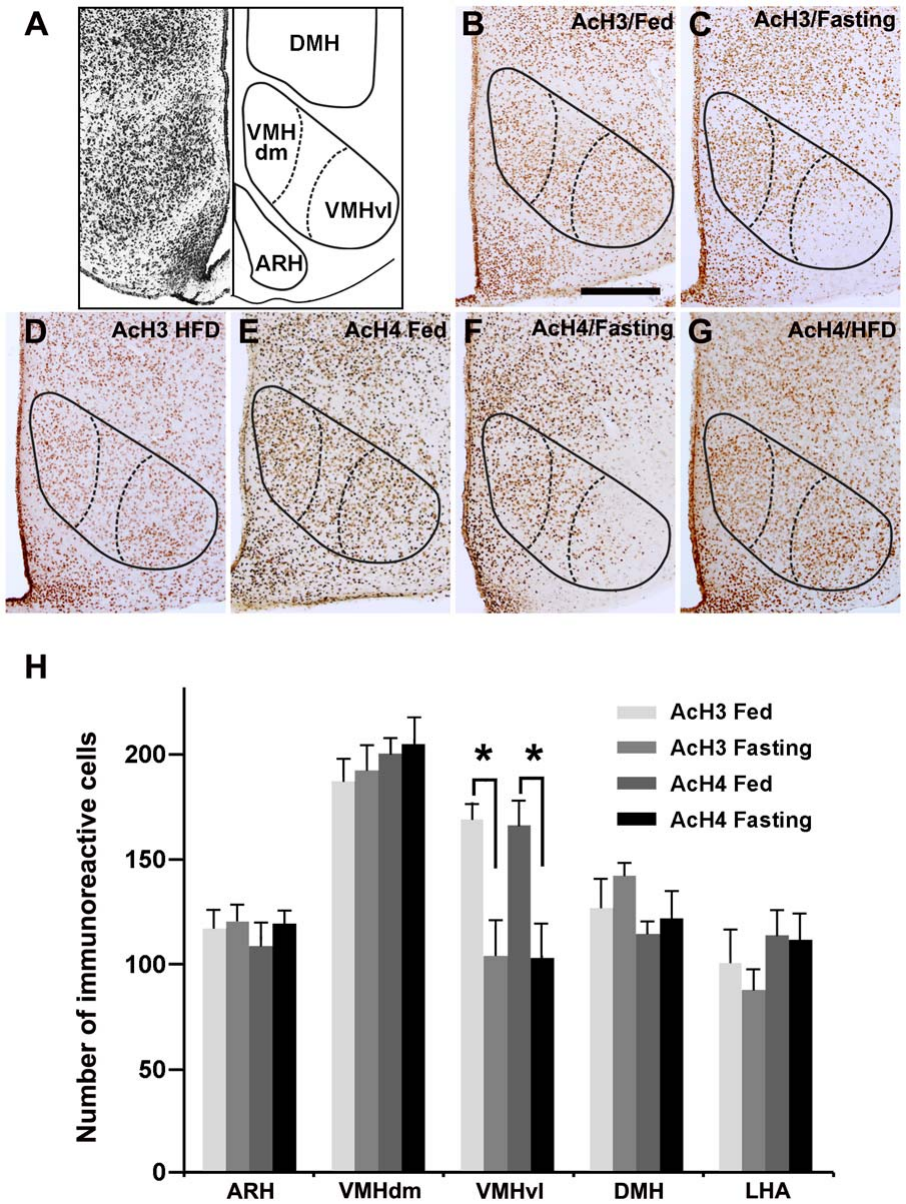
Fasting decreases the number of acetylated histone-positive cells in the VMHvl. A) Schematic illustration of the ARH, VMHdm, VMHvl, and DMH (right) and Nissl-stained section (left) of the coronal section of the medial hypothalamus. The third ventricle is in the midline. B–G) Immunoreactive cells for acetylated histone H3 on lysine 14 (AcH3; B, C, D) and acetylated histone H4 on lysine 12 (AcH4; E, F, G) under fed (B, E), fasting (C, F), and high-fat feeing (D, G), were visualized using DAB. The third ventricle is to the left. In mice fed a low-fat diet (B, E) or a high-fat diet (D, G), most cells in the ARH, VMH, and DMH were positive for AcH3 (B, D) and AcH4 (E, G). In fasting mice, a small numbers of cells in the VMHvl were positive for AcH3 (C) and AcH4 (F). H) A graph showing the number of AcH3- and AcH4-positive cells in the ARH, VMHdm, VMHvl, DMH, and LHA. The numbers of AcH3- and AcH4-positive cells in the VMHvl of fasting mice were significantly smaller than those of fed mice (n = 6). Scale bar: 250 µm. *P<0.05.

Next, we performed histological examination of histone acetylation using antibodies for histone H3 acetylated on lysine 14 (AcH3) and histone H4 acetylated on lysine 12 (AcH4). For both AcH3 and AcH4 immunostaining, large numbers of strongly positive cells were observed throughout the brain, including in the medial hypothalamus (Figure 5B, E). After 16 hours of fasting, a reduced number of cells positive for AcH3 and AcH4 were found in the VMHvl and in the adjacent lateral region of the VMHvl, whearas the number of AcH3- and AcH4-positive cells did not change in the VMHdm (Figure 5C, F, H) and the anterior subdivision of VMH (105±14, 101±11, and110 ±15 for fed, fasting, and high-fat fed mice, respectively). A high-fat diet did not alter the number of AcH3- and AcH4-positive cells in any of the hypothalamic nuclei (Figure 5D, G).

**Figure 6:**
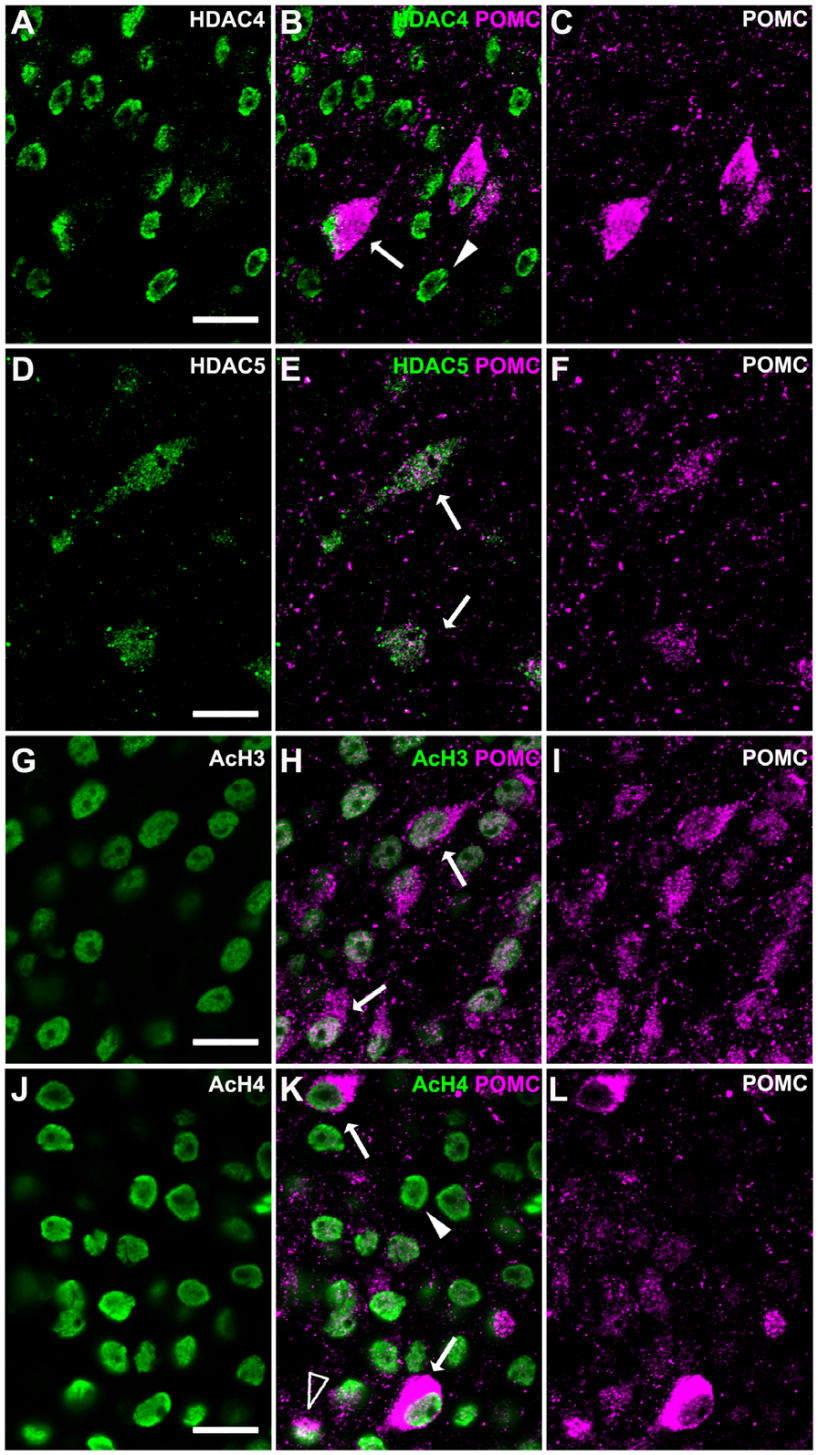
Expressions of HDAC4, −5, AcH3, and AcH4s in POMC neurons in the ARH. A, B, C) HDAC4-positive nuclei were found in POMC-positive cells (arrow in B) and POMC-negative cells (arrowhead in B) in the ARH of a high-fat fed mouse. D, E, F) HDAC5-immunoreactivities were found in the cytoplasm of POMC-positive cells (arrows in E) of a fed mouse. G, H, I) AcH3-positive nuclei were found in POMC-positive cells (arrows in H) of a fed mouse. J, K, L) AcH4-immunoreactivities were found in the nuclei of strongly POMC-positive cells (arrows in K), scattered moderate POMC-positive cells (open arrow in K), and POMC-negative cells (arrowhead in K) of a high-fat fed mouse. Scale bars: 50 µm.

To examine whether a specific cell group in the ARH changes the expression of HDACs or histone acetylation under different metabolic status, we performed double immunofluorescence of POMC with HDAC4, −5, AcH3, and AcH4, which were immunoreactive in the ARH as described above. Confocal microscopy showed that all POMC neurons were positive for HDAC4 (Figure 6A,B,C; 100 out of 100 POMC-positive cells), AcH3 (Figure 6G,H,I; 100/100), and AcH4 (Figure 6J,K,L; 100/100) under fed condition as well as fasting or high-fat feeding condition. HDAC5-immunoreactivities were detected in most POMC neurons of fed mice (Figure 6D,E,F; 95±4/100). Similarly, HDAC5-immunoreactivites in most POMC neurons were found in fasting mice (94±8/100) and mice on a high-fat diet (95±4/100).

## Discussion

The present study shows that the expression profile of HDAC family members is altered in response to fasting and high-fat diet-induced obesity. Fasting increased *HDAC3* and *−4* levels and decreased *HDAC10* and *−11* levels in the medial hypothalamus, whereas a high-fat diet increased *HDAC5* and *−8* levels. Furthermore, fasting decreased the number of AcH3- and AcH4-positive cells in the VMHvl.

It is well established that fasting modifies the expression of a variety of proteins, including POMC, AgRP and thyrotropin-releasing hormone (TRH) to result in altered behavior [19], [29]. Behavioral changes include increased locomotor activity, decreased anxiety behaviors, and suppressed sexual and reproductive behaviors [16], [30]. These behavioral changes encourage animals to explore for food and to utilize the appropriate amount of energy necessary to survive and to keep body weight stable [31]. Although fasting alters the gene expression profile of the hypothalamus via several signal transduction pathways [20]–[22], the role of HDACs in the gene expression of the hypothalamus has not been clarified. Increased *HDAC3* and *−4* expression in response to fasting suggests that HDAC3 and −4 are involved in the modulation of gene expression in the medial hypothalamus. HDAC3 and −4 form a complex with the transcriptional corepressors N-CoR/SMART in the cell nucleus to suppress gene expression [25], [32]. The deacetylase activity of HDAC3 and the nucleo-cytoplasmic localization of HDAC4 are regulated by both phosphorylation and dephosphorylation, processes which involve proteins such as Ca^2+^/calmodulin-dependent kinases (CaMKs) and protein phosphatases [25], [33]. *HDAC10* and *−11* levels are high in the medial hypothalamus and decrease after fasting. Previous work has found that HDAC10 associates with HDAC3 and −4 [34]–[36]. Thus, the increased expressions of HDAC3 and −4, along with the reduced expression of HDAC10, may result in altered expression of genes related to feeding behavior.

HDAC8 was expressed in a subset of neurons in the anterior parvicellular and periventricular subdivisions of the PVH and the expression level was decreased upon fasting. Interestingly, the anterior parvicellular and periventricular subdivisions of the PVH are rich in neurons expressing TRH [37] whose expression was decreased upon fasting [29], and a recently identified satiety neuropeptide, nesfatin−1 [38]. This suggests the possibility that HDAC8 may link energy condition to gene expression change of a subset of PVN neurons. Future studies will be required to characterize HDAC8-high expressing cells and to elucidate the role of HDAC8 in animal behavior.

We did not find any change in HDACs- or acetylated histone-immunoreactivities of the whole ARH as well as POMC neurons in response to fasting or a high-fat diet. However, it is important to note that our findings do not exclude altered histone acetylation of the promoter region of the POMC gene in POMC neurons, because an antibody for acetylated histone detects general histone acetylation status, not that of a specific gene.

Our immunohistochemical study also determined that fasting decreased the histone acetylation status in the VMHvl, which plays a crucial role in aggression and reproductive behaviors [14], [39]–[42]. Increased expressions of HDAC3, and −4 in the medial hypothalamus during fasting suggest the role of HDAC3 and −4 in promoting deacetylated status in the VMHvl, resulting in altered gene expression and function.

## Materials and Methods

### Animals

C57BL/6J male mice were used in this study. Mice were provided food and water *ad libitum*, maintained on a 12-hour light/dark cycle and housed under controlled temperature and humidity conditions. All procedures were approved by the Institutional Animal Care and Use Committee of Toho University (Approved protocol ID #10-51-81). To examine the effects of fasting, mice were removed from food at Zeitgeber time 20 (ZT20) and sacrificed at ZT12. Another group of mice was fed a high-fat diet (D12451; Research Diet) starting at 8 weeks of age. A low-fat diet, or normal chow, (MF; Oriental Yeast) provided 3.6 kcal/g of energy (61% carbohydrate, 26% protein, and 13% fat), whereas a high-fat diet provided 4.7 kcal/g of energy (35% carbohydrate, 20% protein, and 45% fat). After 4-week of high-fat feeding, mice were sacrificed at ZT12.

### Quantitative PCR

Mice were sacrificed by cervical dislocation while deeply anesthetized using sodium pentobarbital. Then, the brain was rapidly removed and the medial hypothalamus was dissected on ice, rostrally at the optic chiasm, caudally at the mammillary bodies, 1 mm bilateral from the midline and 1.5 mm dorsal of the ventral surface. This dissected tissue included the ARH, VMH, DMH, PVH, anterior hypothalamic area, and a part of the LHA. Total RNA was isolated using the RNeasy Lipid Tissue Mini kit (Qiagen, Chatsworth, CA) and used for cDNA synthesis with oligo dT primers and a PrimeScript reverse transcriptase kit (TaKaRa Bio, Otsu, Japan). Real-time quantitative PCR reactions were performed on cDNA with ABI Prism 7000 Sequence Detection System using SYBR GREEN PreMix Ex Taq (TaKaRa) according to the manufacturer's manual. The number of cycles required to reach a threshold fluorescence level was scored and used for generating standard curves and interpolating mRNA concentration levels. A relative quantification method was employed for quantification of target molecules by calculating the ratio between the amount of the target molecule and a reference molecule within the same sample, according to the manufacturer's protocol. Amplification of a single PCR product was confirmed by monitoring the dissociation curve. The averages of glyceraldehyde-3-phosphate dehydrogenase (*GAPDH*) mRNA and *β-actin* mRNA levels were used for normalization. Primer sets used included 5-tgcgtggaaagaaaacaacc-3 and 5-aagcctgaaaaggggtccta-3 for *HDAC1*, 5-taccacatgcacctggtgtt-3 and 5-tttgtctgatgctcgaatgg-3 for *HDAC2*, 5-cccgaggagaactacagcag-3 and 5-actcttggggacacagcatc-3 for *HDAC3,* 5-tctgccaaatgttttgggta-3 and 5-tcacagatggctgtcaggtc-3 for *HDAC4,* 5-gggatattcaccatggcaac-3 and 5-ctccaccaacctcttcagga-3 for *HDAC5,* 5-agcctcgcatacaaacaagc-3 and 5-tcagaatcatgggcttcctc-3 for *HDAC6,* 5-tgtccagactcctggctacc-3 and 5-catgggttcttcctcttcca-3 for *HDAC7,* 5-ggctcgttgctggacatact-3 and 5-ccagcacataatcaggacca-3 for *HDAC8,* 5-gcagcagatccacatgaaca-3 and 5-agaggctgctctgtcttcca-3 for *HDAC9,* 5-tgcttacctcctggagtgct-3 and 5-gtgtggcaagatcctcatcc-3 for *HDAC10,* 5-ggagaggaatgtcaggaggtc-3 and 5-ccacttcatccctcttcacaa-3 for *HDAC11,* 5-ggcctcaagaagacaactgc-3 and 5-gactcgtgcagccttacaca-3 for *Agrp,* 5-gacggccaggtcatcactat-3 and 5-cggatgtcaacgtcacactt-3 for *β-actin, and* 5-agaacatcatccctgcatcc-3 and 5-cacattgggggtaggaacac-3 for *GAPDH.*

### Immunohistochemistry

Animals were deeply anesthetized with sodium pentobarbital and perfused transcardially with phosphate-buffered saline (PBS) followed by phosphate-buffered 4% paraformaldehyde (PFA). Brains were removed, post-fixed overnight in phosphate-buffered 4% PFA and equilibrated in 30% sucrose for two days. Brains were sectioned on a cryostat at 35 µm. Sections were stored in a cryoprotective tissue collection solution (25% glycerol, 30% ethylene glycol, 0.05 M phosphate buffer) at -20°C until use. Immunohistochemistry using immunoperoxidase was performed using a free-floating method. After pretreated with 0.3% hydrogen peroxide in PBS for 15 minutes, sections were preincubated for 1 hour in blocking solution (0.1 M phosphate buffer, 0.25% Triton X-100, and 5% normal goat serum). Brain sections were incubated with primary antibodies for histone H3 acetylation on lysine 14 (AcH3, 1∶3000, rabbit monoclonal, 04-1044, Millipore, Billerica, MA), histone H4 acetylation on lysine 12 (AcH4, 1∶10,000, rabbit monoclonal, 04-119, Millipore), HDAC3 (1∶100, rabbit monoclonal, ab32369, Abcam, Cambridge, MA), HDAC4 (1∶100, rabbit polyclonal, ab79521, Abcam), HDAC5 (1∶100, rabbit polyclonal, ab55403, Abcam), HDAC8 (1∶250, rabbit polyclonal, ab39664, Abcam), HDAC10 (1∶50, rabbit polyclonal, ab53096, Abcam), and HDAC11 (1∶100, rabbit polyclonal, ab18973, Abcam) followed by incubation with biotinylated anti-rabbit IgG (1∶400, BA1000, Vector Laboratories, Burlingame, CA). Sections were then incubated in avidin-biotin-horseradish peroxidase conjugate (Elite ABC kit, Vector). Immunoreactivity was visualized using 3,3'-diaminobenzidine (DAB). Images were captured by a CCD camera (DP70, Olympus, Tokyo, Japan) at a 40 x and 100 x magnification using DP controller software (Olympus). Immunofluorescence was also performed using a free-floating method. After preincubated for 1 hour in blocking solution (0.1 M phosphate buffer, 0.25% Triton X-100, and 5% normal donkey serum), brain sections were incubated with primary antibodies for POMC (1∶150, goat polyclonal, ab32893, Abcam),and with antibodies for AcH3 (1∶3000), AcH4 (1∶10,000), HDAC4, (1∶100), or HDAC5 (1∶100) in 3% normal donkey serum and 0.25% Triton X-100 overnight at 4°C. After washing in PBS, sections were incubated with Alexa 555-conjugated anti-goat IgG (1∶400, A21432, Invitrogen, Carlsbad, CA), Alexa 488-conjugated anti-rabbit IgG (1∶400, A21206, Invitrogen), and Hoechst33342 (2 µg/ml, H21492, Invitrogen) for 1 hour at room temperature. After several washes, sections were mounted on a glass slide with Gel/Mount (Biomeda, Foster city, CA). Immunofluorescence images were captured by using a scanning confocal microscope (LSM510 META, Zeiss, Oberkochen, Germany), with C-Apochromat 40/1.2 water immersion lens. The images were obtained at 1 µm optical thickness with the frame size of 1,024×1,024 pixels. For each double-label immunohistochemical image, each channel was collected separately with single wavelength excitation and then merged to produce the composite image. Experimental controls were prepared in which one or both of the primary antibodies were omitted from the reaction solution. Confocal laser scanning microscopy showed no immunolabeling of omitted antibodies in the control sections. Photoshop CS3 (Adobe Systems, Mountain View, CA) was used to combine drawings and digital images into plates. The contrast and brightness of images were adjusted. Red-green fluorescence images were converted to magenta-green for the readers who are color blind. Areas of overlap will appear white.

### Image analysis

To count the number of cells positive for immunoperoxidase reaction, we captured areas of 1.8 mm×1.35 mm (1360×1024 pixels) from coronal hypothalamic sections by a CCD camera (DP70, Olympus) using 10x objective lens and DP controller software. Microscopic images were captured at the focal plane in which the maximum number of immunoreactive cells was recognized. The captured images were processed using Image J software (National Institutes of Health). After running threshold and watershed tools, particles with the size between 100–1000 pixels were counted. The area for positive cell count was 250 µm x 250 µm, except for ARH, for which the count area was 150 µm x 150 µm. Immunoreactive cells from two sections per animal (5–6 mice per group) were counted by observers blinded to the animal's group. The average number of cells counted in each mouse was taken for statistical comparisons.

To assess the double immunofluorescence data of the ARH, we randomly selected 100 of POMC-positive cells in the ARH from 3–5 optical views per animal (5 mice per group). The percentage of double-labeled cells was determined.

### Data Analysis

Data are presented as means ± standard error of the mean (SEM). mRNA expression levels were determined by quantitative PCR, and the number of positive cells on the immunohistochemical examination was assessed using one-way analysis of variance followed by a Tukey's *post hoc* test.
